# Fermented dairy products intake and stroke risk: analyses of NHANES 2007–2018 data

**DOI:** 10.3389/fnut.2025.1593174

**Published:** 2025-07-21

**Authors:** Sijia Ma, Yongyue Miao, Xian Wu

**Affiliations:** ^1^Heilongjiang University of Chinese Medicine, Harbin, China; ^2^First Affiliated Hospital of Heilongjiang University of Chinese Medicine, Harbin, China

**Keywords:** NHANES, stroke, fermented dairy products, yogurt, adult

## Abstract

**Background:**

Stroke ranks among the main diseases resulting in death and disability, imposing a heavy burden on both the country and individuals. Healthy foods can effectively prevent the occurrence of stroke, and fermented dairy products are among them. However, in previous studies, the correlation between stroke and fermented dairy products remains controversial.

**Methods:**

The intake of fermented dairy products and the identification of stroke both originated from the data of the NHANES database from 2007 to 2018. This study used a weighted regression model to analyze the association between the total intake of fermented dairy products and the intake of various types of fermented dairy products (yogurt, cheese and buttermilk) and stroke, and conducted subgroup analyses and interaction tests.

**Results:**

This study included 27,487 American adults, of whom 2.9% had suffered from a stroke. The results of regression analysis indicated that total intake of fermented dairy products and yogurt intake were negatively correlated with stroke. For total intake, after adjusting for all confounding variables, the results revealed that every 50 g rise in intake led to a 7% decline in the stroke risk (OR = 0.93, 95% CI: 0.88–1.00). Meanwhile, when compared to participants having no consumption of fermented dairy products, those with a low intake had a 21% lower probability of stroke (OR = 0.79, 95% CI: 0.66–0.95). Subgroup analysis showed that smoking interacted with stroke and fermented dairy products (*p* = 0.047). For yogurt, after adjusting for all confounding variables, the results indicated that for every 50 g rise in intake, the probability of stroke declined by 7% (OR = 0.93, 95% CI: 0.86–1.00). However, only high intake of yogurt was associated with a protective effect against stroke, and this relationship remained stable across three models (Model I: OR = 0.49, 95% CI: 0.33–0.75; Model II: OR = 0.50, 95% CI: 0.33–0.75; Model III: OR = 0.62, 95% CI: 0.40–0.96). In contrast, no significant associations were found between cheese and buttermilk intake and stroke risk.

**Conclusion:**

This study discovered that, among American adults, the total quantity of fermented dairy products as well as yogurt had an inverse correlation with the risk of stroke, while this correlation did not exist for cheese or buttermilk.

## 1 Introduction

Stroke is a disease primarily characterized by symptoms of ischemic or hemorrhagic brain injury, posing a severe threat to human health and ranking as the third leading cause of death after ischemic heart disease and COVID-19 (1). According to the latest data, the incidence and prevalence of stroke have declined globally between 1990 and 2021 (2). However, the incidence among younger populations is on the rise (3). Meanwhile, stroke-attributable deaths and disabilities have increased by 43 and 32% (2), respectively. Due to its elevated mortality and disability levels, stroke persists in placing a substantial economic strain on both society and families. Studies predict that by 2050, the burden of stroke will increase by 81%, with a more significant rise in upper-middle-income countries (4). Therefore, preventing stroke is of utmost importance.

To prevent the occurrence of stroke, it is essential to identify the inducing factors of stroke. Although there are numerous contributing factors, in recent years, the theory that explains the occurrence of stroke from the perspective of the brain-gut microbiota mechanism has received widespread attention (5). There exists a bidirectional communication between the brain and the gut. When the gut microbiota is dysbiotic, it can trigger the abnormal emission of bacterial metabolites or neurotransmitters in the intestine. These substances can be transmitted via the vagus nerve or directly enter the bloodstream and cross the blood–brain barrier, thereby affecting the neurovascular system and triggering a stroke (6). Furthermore, the gut microbiota can also indirectly have an effect on the happening of stroke by exerting an influence on pathogenic factors including body weight, blood pressure, and blood glucose levels (7). Food is a direct factor influencing the gut microbiota. As an effective carrier of probiotics, fermented dairy products have a significant impact on the gut microenvironment, making them an indispensable part of the diet. As a traditional food, fermented dairy products not only appear in the food guidelines of many countries (8) but also in the recommended dietary patterns for stroke prevention, such as the Mediterranean diet (9). Compared with milk, fermented dairy products not only contain all the nutrients of milk, but some components, such as proteins and minerals, are present in higher concentrations than in milk (10). Moreover, the probiotics generated during the fermentation process can make the raw materials more easily absorbed by transforming their chemical compositions (11). Meanwhile, the fermentation process breaks down the lactose in dairy products, making them suitable for consumption by people with lactose intolerance (12), thus expanding the group of beneficiaries of dairy products. A study from Sweden (13) found that an appropriate intake of fermented dairy products (400–600 g/day) can lower the probability of death (HR = 0.93, 95% CI: 0.87–0.99), while a high-dose intake of non-fermented dairy products may increase the risk of death (HR = 1.34, 95% CI: 1.14–1.59). Evidently, fermented dairy products are beneficial to our health.

The link between fermented dairy products and stroke has been documented in previous reports. As early as in the 1980s, a study on adult women found that consuming yogurt could reduce the risk of stroke, and the same relationship also existed for hard cheese (14). A cohort study involving 74,138 participants found that the intake of a small amount of fermented dairy products could reduce the incidence of stroke (RR = 0.89, 95% CI: 0.81–0.98), but there was no association between non-fermented dairy products and the risk of stroke (15). A meta-analysis of prospective cohort studies showed that fermented milk (RR = 0.80, 95% CI: 0.71–0.89) and cheese (RR = 0.94, 95% CI: 0.89–0.995) were both tied to a lower incidence of stroke, but this relationship did not exist for non-fermented milk (RR = 1.02, 95% CI: 0.89–1.17) (16). Thus, it is evident that fermented dairy products are negatively correlated with the occurrence of stroke and can be considered a healthy dietary option for stroke prevention.

However, a study targeting individuals aged 55 and above found no significant correlation between stroke and fermented dairy products (17). Similarly, Susanna et al. (18) conducted a 10-year follow-up study involving 74,961 individuals from Sweden. The results showed that after adjusting for all variables, the negative correlation between yogurt or cheese and stroke disappeared. Another study from Sweden reached a similar conclusion (19). In contrast to the aforementioned studies, a study including 26,566 men found that yogurt consumption may increase the risk of subarachnoid hemorrhage (RR = 1.83, 95% CI: 1.20–2.0) (20). Additionally, a meta-analysis pointed out that most of the existing research into the connection of yogurt or cheese with stroke risk centers around comparing low and high intake amounts, but a clear dose–response relationship has not yet been established (21). It is evident that the relationship between fermented dairy products and stroke remains controversial. The association between different types of fermented dairy products and stroke is not well-defined, and most relevant studies have been conducted in European countries. Only 0.1% of Americans have healthy eating habits according to data (22), while dietary modifications are of vital significance in the primary prevention of stroke (23). Therefore, it is particularly urgent to clarify the association between fermented dairy products and stroke among American adults.

This study analyzed the National Health and Nutrition Examination Survey (NHANES) data in order to clarify the association between total fermented dairy product intake and individual fermented dairy product intake (yogurt, cheese and buttermilk) with stroke among adult Americans.

## 2 Methods

### 2.1 Study population

NHANES is a cross-sectional survey organized and carried out biennially by the Centers for Disease Control and Prevention (CDC) of the United States. This database adopts a sophisticated sampling design and contains comprehensive information, enabling it to representatively mirror the health and nutritional conditions of Americans.

**Figure 1:**
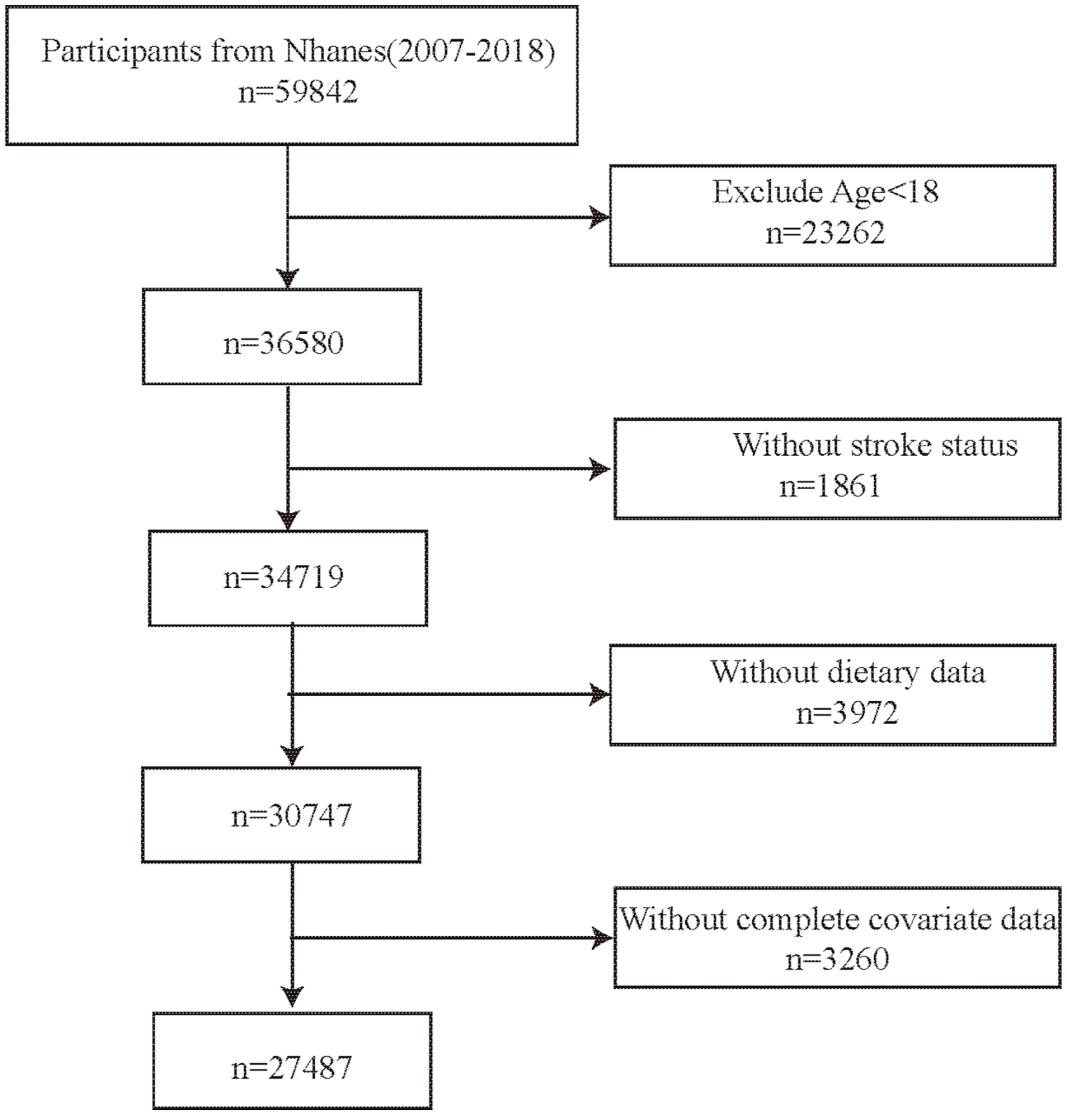
Flow chart.

In the study, six NHANES cycles from 2007 to 2018 were selected. The inclusion criteria were as follows: (1) age ≥18 years, (2) participants with complete stroke status, (3) participants with complete dietary data (4) participants with complete information on other confounding factors. Finally, this survey included 27,487 participants (Figure 1).

### 2.2 Definition of stroke

The primary outcome was ascertained using the Medical Conditions Questionnaire administered via face-to-face interviews. During these interviews, participants were specifically asked whether they had ever been diagnosed with a stroke. Those who answered affirmatively were categorized as having defined with stroke.

### 2.3 Fermented dairy products intake

The intake amount of fermented dairy products was obtained from the 24-h dietary recall interview. Each participant provided information on the types, weights, times, occasions, and other relevant details of the foods or beverages they consumed. Different foods and beverages were assigned corresponding codes. Querying the codes provided on the website revealed that the database only included three types of fermented dairy products: yogurt, cheese, and buttermilk. It should be noted that buttermilk products included in the analysis were specifically cultured buttermilk, as defined by their fermentation-based production process. Therefore, this study separately calculated the weights of these three fermented dairy products consumed by participants and summed them up to derive the total intake. Ultimately, the mean consumption quantity of different fermented dairy products obtained from two 24-h dietary recall surveys served as the foundation for analysis. Those participants without records of these codes were defined as non-consumers. After the statistics were compiled, the participants were separated into three groups based on their median intake of each fermented dairy product: (1) Non-consumers; (2) low-intake group (0—median value); (3) high-intake group (> median value).

### 2.4 Covariates

Referring to relevant articles (24–26), we selected 15 confounding variables from the database. Among them, gender, race, age, educational attainment, drinking (having at least 12 alcohol drinks peer year or not), smoking (smoking cigarettes now or not), physical activity (having work activity or recreational activity), hypertension, diabetes, cardiovascular disease (CVD) and medication use were all obtained from face-to-face interviews. CVDs included conditions such as coronary heart disease, congestive heart failure, heart attack, and angina. It should be noted that CVD mentioned in this study did not include stroke, as stroke was the outcome measure. Medication use included participants’ current use of anti-hypertensive drug, anti-lipemic drug, and anti-diabetic medication (insulin or diabetic pills). Body mass index (BMI) was from physical examination and categorized into three groups: (1) normal weight (BMI = 18.5–24.9) (2) underweight (BMI < 18.5), (3) overweight (BMI ≥ 25.0). For the diagnosis of hyperlipidemia, in addition to the information obtained from the questionnaire survey, it also includes those who meet any one of the following blood test results (27): (1) total cholesterol ≥200 mg/dL, (2) triglycerides ≥150 mg/dL, (3) high-density lipoprotein ≤50 mg/dL, (4) low-density lipoprotein ≥130 mg/dL. Additionally, participants’ cow milk consumption was included as a covariate, which was derived from 24-h dietary recalls.

### 2.5 Statistical analysis

To guarantee that the results accurately represented the American civilian population, each participant was allocated a sample weight when they accepted the survey. Based on the official website descriptions and analytical standards, weight was also taken into account during data analysis. The data was organized and analyzed using R software (version 4.4.2).^1^ The study used a two-tailed *p*-value of <0.05 for statistical significance.

In baseline data analysis, the median and quartiles were used to describe continuous data, and non-parametric test was used to analyze differences; in contrast, percentages were used to describe categorical data, and chi-square tests were used to evaluate differences. Multivariate regression analysis was employed across three distinct models to evaluate the association between fermented dairy intake and stroke risk. Model I was unadjusted for any covariates. Model II was adjusted for sex, age, and race. Model III was fully adjusted for all potential confounding variables included in the study. Then, fermented dairy products were treated as categorical variables in the regression analysis. These analyses were carried out four times, with the categories of total amount, yogurt, cheese and buttermilk.

Subsequently, subgroup analyses were conducted based on sex, race, education attainment, smoking, drinking, BMI, physical activity, diabetes, hypertension, hyperlipidemia, CVD and medication use to evaluate the association between fermented dairy product consumption and stroke risk in specific population subgroups. Statistical interaction tests were also performed to assess potential effect modification by these demographic factors.

Additionally, to explore whether fat content in fermented dairy products affects stroke occurrence, this study also conducted regression analysis between fat content and stroke. This analysis was performed separately in subgroups of individuals consuming yogurt, buttermilk, and cheese.

## 3 Results

### 3.1 Baseline characteristics

The study ultimately included 27,487 participants, among whom 1,055 had experienced a stroke. Participants with a habit of consuming fermented dairy products accounted for 59.8% of the total population. Yogurt consumers made up 14.8%, cheese consumers accounted for 54.3%, and only 0.2% of the participants consumed buttermilk. Due to the low prevalence of buttermilk consumption, the median (intake) of buttermilk was zero. Overall, as shown in Table 1, the risk of stroke was significantly associated with age (*p* < 0.001), education (*p* < 0.001), race (*p* < 0.001), PIR (*p* < 0.001), drinking (*p* = 0.011), smoking (*p* = 0.008), BMI (*p* = 0.002), Physical activity (*p* < 0.001), hypertension (*p* < 0.001), diabetes (*p* < 0.001), hyperlipidemia (*p* = 0.001), CVD (*p* < 0.001), anti-hypertensive drug (*p* < 0.001), anti-diabetic medication (*p* < 0.001) and anti-lipemic drug (*p* < 0.001). Compared with participants without stroke, those who had a stroke were older, had lower educational attainment, had a higher prevalence of smoking, had a higher proportion of Black people, had a lower proportion of Mexican Americans, had lower income levels and had a lower prevalence of physical activity. Additionally, these individuals had a higher likelihood of being overweight, along with a more widespread prevalence of hypertension, diabetes, hyperlipidemia, and CVD. Similarly, a higher proportion of individuals with stroke were on medications. However, the study found that participants without stroke were more likely to have a habit of alcohol consumption. This study also found no association between stroke and sex.

**Table 1:** Baseline characteristics, weighted.

Characteristic	Overall (*n* = 27,487)	No stroke (*n* = 26,432)	Stroke (*n* = 1,055)	*p*
Age (years)	49.00 (36.00, 61.00)	49.00 (35.00, 61.00)	68.83 (57.64, 79.78)	<0.001
Sex (%)				0.109
Male	48.1	48.2	44.5	
Female	51.9	51.8	55.5	
Education (%)				<0.001
Less than 9th grade	4.7	4.6	9.1	
9–11th grade	10.1	10.0	15.8	
High school graduate	23.1	22.8	32.8	
Some college or AA degree	32.1	32.3	24.2	
College graduate or above	30	30.3	17.8	
Race (%)				<0.001
Non-Hispanic White	67.5	67.5	68	
Non-Hispanic Black	10.8	10.7	16.3	
Mexican American	8.2	8.3	4.7	
Other	13.4	13.5	11.0	
PIR	3.92 (2.06, 5.00)	3.97 (2.10, 5.00)	2.43 (1.21, 3.67)	<0.001
Drinking (%)				0.011
No	22.9	22.7	27.9	
Yes	77.1	77.3	72.1	
Smoking (%)				0.008
No	80.2	80.3	75.3	
Yes	19.8	19.7	24.7	
BMI (kg/m^2^)	27.00 (23.69, 31.41)	27.00 (23.64, 31.40)	27.90 (24.88, 33.65)	0.002
BMI group (%)				0.037
Normal weight	28.1	28.3	23.3	
Underweight	1.6	1.6	1.7	
Overweight	70.3	70.1	75.0	
Physical activity (%)				<0.001
No	21.4	21.7	45.0	
Yes	78.6	79.3	55.0	
Hypertension (%)				<0.001
No	68.0	69.2	27.1	
Yes	32.0	30.8	72.9	
Diabetes (%)				<0.001
No	90.6	90.9	68.4	
Yes	9.4	9.1	31.6	
Hyperlipidemia (%)				<0.001
No	33.1	33.5	19.4	
Yes	66.9	66.5	80.6	
CVD (%)				<0.001
No	93.3	94.2	64.1	
Yes	6.7	5.8	35.9	
Anti-hypertensive drug (%)				<0.001
No	76.6	77.8	36.3	
Yes	23.4	22.2	63.7	
Anti-diabetic medication (%)				<0.001
No	92.0	92.5	75.4	
Yes	8.0	7.5	24.6	
Anti-lipemic drug (%)				<0.001
No	82.3	83.2	51.9	
Yes	17.7	16.8	48.1	
Cow milk intake (g/day)	15.25 (0, 244.00)	16.41 (0, 244.00)	5.82 (0, 182.50)	0.649
Total intake of fermented dairy products (g/day)	185.39 (145.19, 245.00)	186.02 (145.48, 245.00)	170.00 (139.10, 195.20)	0.002
Fermented dairy products (%)				<0.001
0 g/day	40.2	39.9	50.1	
0–38.3 g/day	28.5	28.6	24.5	
>38.3 g/day	31.3	31.5	25.4	
Yoghurt intake (g/day)	170 (122.50, 214.38)	170 (122.50, 214.38)	150.00 (122.50, 170.25)	0.043
Yoghurt intake (%)				0.004
0 g/day	85.2	85.0	89.8	
0–170	6.9	6.9	6.0	
>170	7.9	8.1	4.2	
Cheese intake (g/day)	18.00 (0, 36.79)	18.62 (0, 37.50)	8.14 (0, 28.25)	0.032
Cheese intake (%)				0.001
0 g/day	45.7	45.4	54.3	
0–28.30 g/day	26.0	26.1	22.5	
>28.30 g/day	28.3	28.5	23.2	
Buttermilk intake (g/day)	0 (0, 0)	0 (0, 0)	0 (0, 0)	0.406
Buttermilk intake (%)				0.940
0 g/day	99.8	99.8	99.8	
0–244.5 g/day	0.1	0.1	0.1	
>244.5 g/day	0.1	0.1	0.1	

Measurement data are described using the median (lower quartile, upper quartile), while count data are described using percentages. PIR, poverty income ratio; BMI, body mass index; CVD, cardiovascular disease. 38.3, 170, 28.3 and 244.5 were the median.

### 3.2 Stroke prevalence according to kinds of fermented dairy products

**Figure 2:**
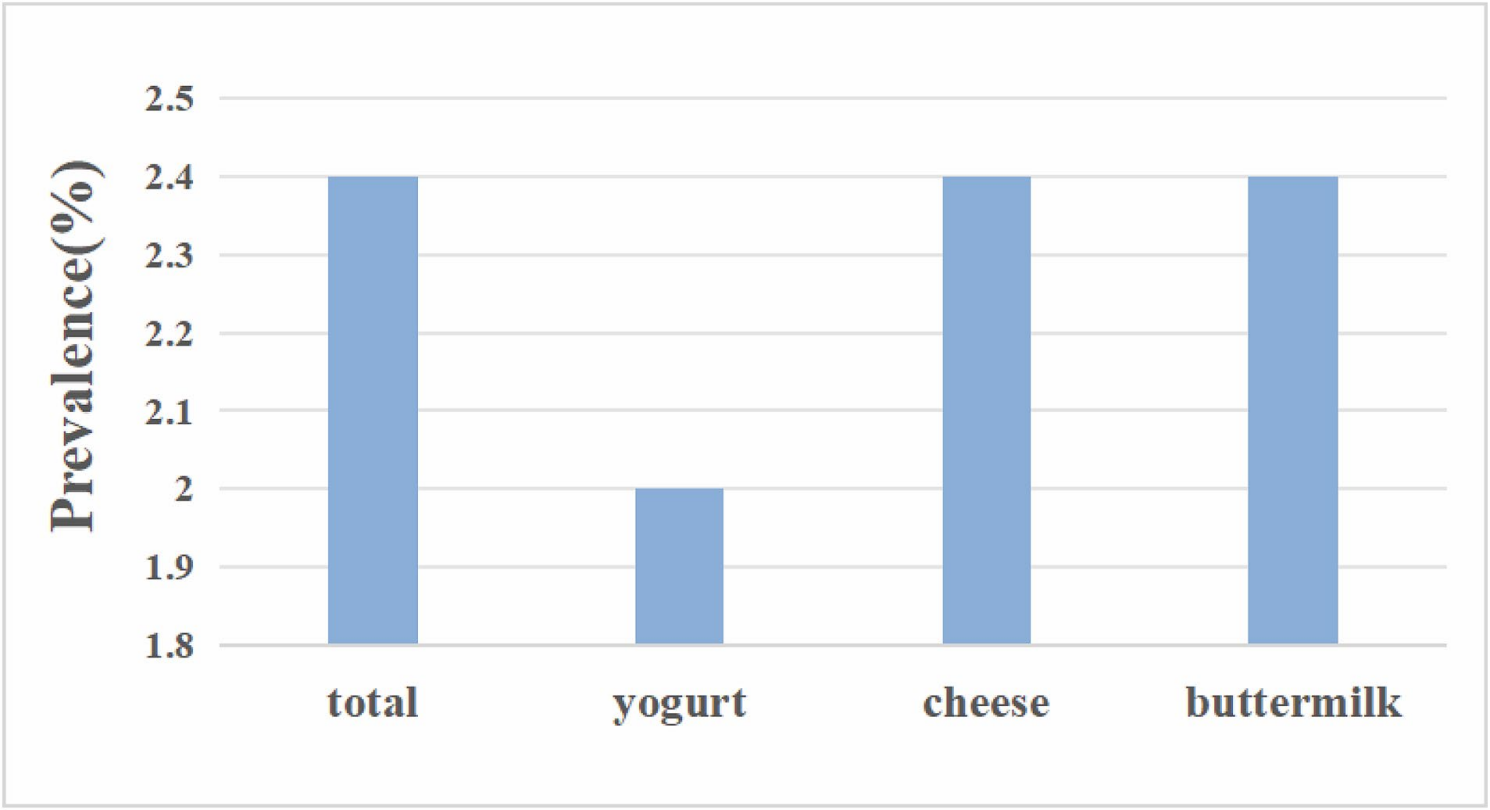
Stroke prevalence according to kinds of fermented dairy products.

This study calculated the stroke incidence rates among populations consuming different types of fermented dairy products, as shown in Figure 2. The incidence rate of stroke was 2.40% (95% CI: 2.11–2.74) totally, 2% (95% CI: 1.56–2.54) for participants who had yogurt, 2.40% (95% CI: 2.11–2.77) for who ate cheese, and 2.40% (95% CI: 0.49–10.63) for who drank buttermilk.

### 3.3 The association between fermented dairy intake and stroke

This study explored the associations among the overall intake of fermented dairy products, as well as the intakes of yogurt, cheese, and buttermilk, and the incidence of stroke. Initially, the intake of fermented dairy products (total, yogurt and buttermilk) was analyzed as a continuous variable and its unit was changed to 50 g/day. According to the *Dietary Guidelines for Americans* (28), 1 cup of dairy equivalent is approximately 240 ml, and 50 g of fermented milk is roughly to 1/5 cup. When analyzing the association between cheese and stroke, the unit was 20 g/day. Then they were divided into three groups: no intake, low intake and high intake for regression analysis.

For total intake (Table 2), when analyzed as a continuous variable, the results from all three models demonstrated a negative association between intake and stroke risk (Model I: OR = 0.88, 95% CI: 0.82–0.94; Model II: OR = 0.89, 95% CI: 0.82–0.95; Model III: OR = 0.93, 95% CI: 0.88–1.00). When treated as a categorical variable, compared with participants who did not consume fermented dairy products, those with a daily intake of 0–38.3 g (38.3 was the median) had a lower stroke risk, and this relationship was consistent across all three models (Model I: OR = 0.68, 95% CI: 0.58–0.81; Model II: OR = 0.74, 95% CI: 0.62–0.88; Model III: OR = 0.79, 95% CI: 0.66–0.95). For participants with high intake, the association with reduced stroke risk was only observed in Model I (OR = 0.64, 95% CI: 0.58–0.81) and Model II (OR = 0.74, 95% CI: 0.57–0.89), but disappeared after full adjustment in Model III. In both Model I and Model II, we also confirmed that there was a relationship between stroke and the amount of fermented dairy intake shown in the trend test (Model I *p* for trend <0.001, Model II *p* for trend = 0.002).

**Table 2:** Association between total intake of fermented dairy products and stroke, weighted.

Exposure	The percentage of participants	Model I OR (95% CI)	*p*	Model II OR (95% CI)	*p*	Model III OR (95% CI)	*p*
Total intake (50 g/day)		0.88 (0.82, 0.94)	<0.001	0.89 (0.82, 0.95)	0.001	0.93 (0.88, 1.00)	0.040
Total intake (g/day)							
≤0	40.2%	Ref		Ref		Ref	
>0, ≤38.3	28.5%	0.68 (0.58, 0.81)	<0.001	0.74 (0.62, 0.88)	<0.001	0.79 (0.66, 0.95)	0.014
>38.3	31.3%	0.64 (0.52, 0.79)	<0.001	0.74 (0.57, 0.89)	0.004	0.87 (0.69, 1.09)	0.200
*p* for trend		<0.001		0.002		0.145	

Model I: no adjustment. Model II: adjust for age, sex, race. Model III: adjust for age, sex, race, PIR, education, smoking, drinking, BMI, physical activity, diabetes, hypertension, hyperlipidemia, CVD, anti-hypertensive drug, anti-lipemic drug, and anti-diabetic medication and cow milk intake. 38.3 was the median. PIR, poverty income ratio; BMI, body mass index; CVD, cardiovascular disease; OR, odds ratio; CI, confidence interval.

Then, this study analyzed the link between the consumption of yogurt and stroke (Table 3). Similarly, the unit for yogurt intake was also converted to 50 g/day. For continuous variables, the results showed that consuming yogurt could reduce the risk of stroke, and this relationship was consistent across all three models (Model I: OR = 0.87, 95% CI: 0.81–0.94; Model II: OR = 0.87, 95% CI: 0.80–0.94; Model III: OR = 0.93, 95% CI: 0.86–1.00). For categorical variables, only those who consumed more than 170 g (170 was the median) of yogurt per day had a reduced stroke risk (Model I: OR = 0.49, 95% CI: 0.33–0.75; Model II: OR = 0.50, 95% CI: 0.33–0.75; Model III: OR = 0.62, 95% CI: 0.40–0.96). Finally, we also found that there was a relationship between the amount of yogurt intake and the risk of stroke (Model I *p* for tend <0.001, Model II *p* for tend <0.001, Model III *p* for tend = 0.050).

**Table 3:** Association between yogurt intake and stroke, weighted.

Exposure	The percentage of participants	Model I OR (95% CI)	*p*	Model II OR (95% CI)	*p*	Model III OR (95% CI)	*p*
Yogurt intake (50 g/day)		0.87 (0.81, 0.94)	<0.001	0.87 (0.80, 0.94)	<0.001	0.93 (0.86, 1.00)	0.044
Yogurt intake (g/day)							
≤0	85.2%	Ref		Ref		Ref	
>0, ≤170	6.9%	0.82 (0.56, 1.21)	0.3	0.76 (0.51, 1.12)	0.2	1.06 (0.71, 1.57)	0.8
>170	7.9%	0.49 (0.33, 0.75)	<0.001	0.50 (0.33, 0.75)	0.001	0.62 (0.40, 0.96)	0.032
*p* for trend		<0.001		<0.001		0.050	

Model I: no adjustment. Model II: adjust for age, sex, race. Model III: adjust for age, sex, race, PIR, education, smoking, drinking, BMI, physical activity, diabetes, hypertension, hyperlipidemia, CVD, anti-hypertensive drug, anti-lipemic drug, and anti-diabetic medication and cow milk intake. 170 was the median. PIR, poverty income ratio; BMI, body mass index; CVD, cardiovascular disease; OR, odds ratio; CI, confidence interval.

Next, we analyzed the relationship between cheese intake and stroke (Table 4). The results showed that when cheese intake was analyzed as a continuous variable, there was no association between the two (*p* > 0.05). For categorical variables, the negative correlation between stroke and cheese intake was only statistically significant in Model I (participants (0–28.3 g/day): OR = 0.72, 95% CI: 0.59–0.88; participants (>28.3 g/day): OR = 0.68, 95% CI: 0.54–0.86), but this relationship lost statistical significance after adjusting for variables (28.3 was the median). In the trend test, we only found statistical significance in Model I and Model II (Model I *p* for trend <0.001, Model II *p* for trend = 0.049).

**Table 4:** Association between cheese intake and stroke, weighted.

Exposure	The percentage of participants	Model I OR (95% CI)	*p*	Model II OR (95% CI)	*p*	Model III OR (95% CI)	*p*
Cheese intake (20 g/day)		0.84 (0.88, 1.01)	0.078	0.97 (0.91, 1.03)	0.3	0.96 (0.82, 1.11)	0.6
Cheese intake (g/day)							
≤0	45.7%	Ref		Ref		Ref	
>0, ≤28.3	26.0%	0.72 (0.59, 0.88)	0.002	0.77 (0.63, 0.95)	0.015	0.84 (0.67, 1.04)	0.10
>28.3	28.3%	0.68 (0.54, 0.86)	0.001	0.82 (0.64, 1.04)	0.10	0.91 (0.71, 1.15)	0.40
*p* for trend		<0.001		0.049		0.288	

Model I: no adjustment. Model II: adjust for age, sex, race. Model III: adjust for age, sex, race, PIR, education, smoking, drinking, BMI, physical activity, diabetes, hypertension, hyperlipidemia, CVD, anti-hypertensive drug, anti-lipemic drug, and anti-diabetic medication and cow milk intake. 28.3 was the median. PIR, poverty income ratio; BMI, body mass index; CVD, cardiovascular disease; OR, odds ratio; CI, confidence interval.

As shown in Table 5, the association between buttermilk intake and stroke was not statistically significant.

**Table 5:** Association between buttermilk intake and stroke, weighted.

Exposure	The percentage of participants	Model I OR (95% CI)	*p*	Model II OR (95% CI)	*p*	Model III OR (95% CI)	*p*
Buttermilk intake (50 g/day)		0.94 (0.78, 1.13)	0.50	0.85 (0.67, 1.07)	0.20	0.87 (0.71, 1.06)	0.2
Buttermilk intake (g/day)							
≤0	99.8%	Ref		Ref		Ref	
>0, ≤244.5	0.1%	0.82 (0.18, 3.74)	0.8	0.44 (0.10, 1.92)	0.3	0.66 (0.15, 3.19)	0.6
>244.5	0.1%	0.81 (0.11, 6.05)	0.8	0.38 (0.05, 2.86)	0.3	0.32 (0.04, 2.47)	0.3
*p* for trend		0.782		0.227		0.214	

Model I: no adjustment. Model II: adjust for age, sex, race. Model III: adjust for age, sex, race, PIR, education, smoking, drinking, BMI, physical activity, diabetes, hypertension, hyperlipidemia, CVD, anti-hypertensive drug, anti-lipemic drug, and anti-diabetic medication and cow milk intake. 244.5 was the median. PIR, poverty income ratio; BMI, body mass index; CVD, cardiovascular disease; OR, odds ratio; CI, confidence interval.

### 3.4 Subgroup analysis

**Figure 3:**
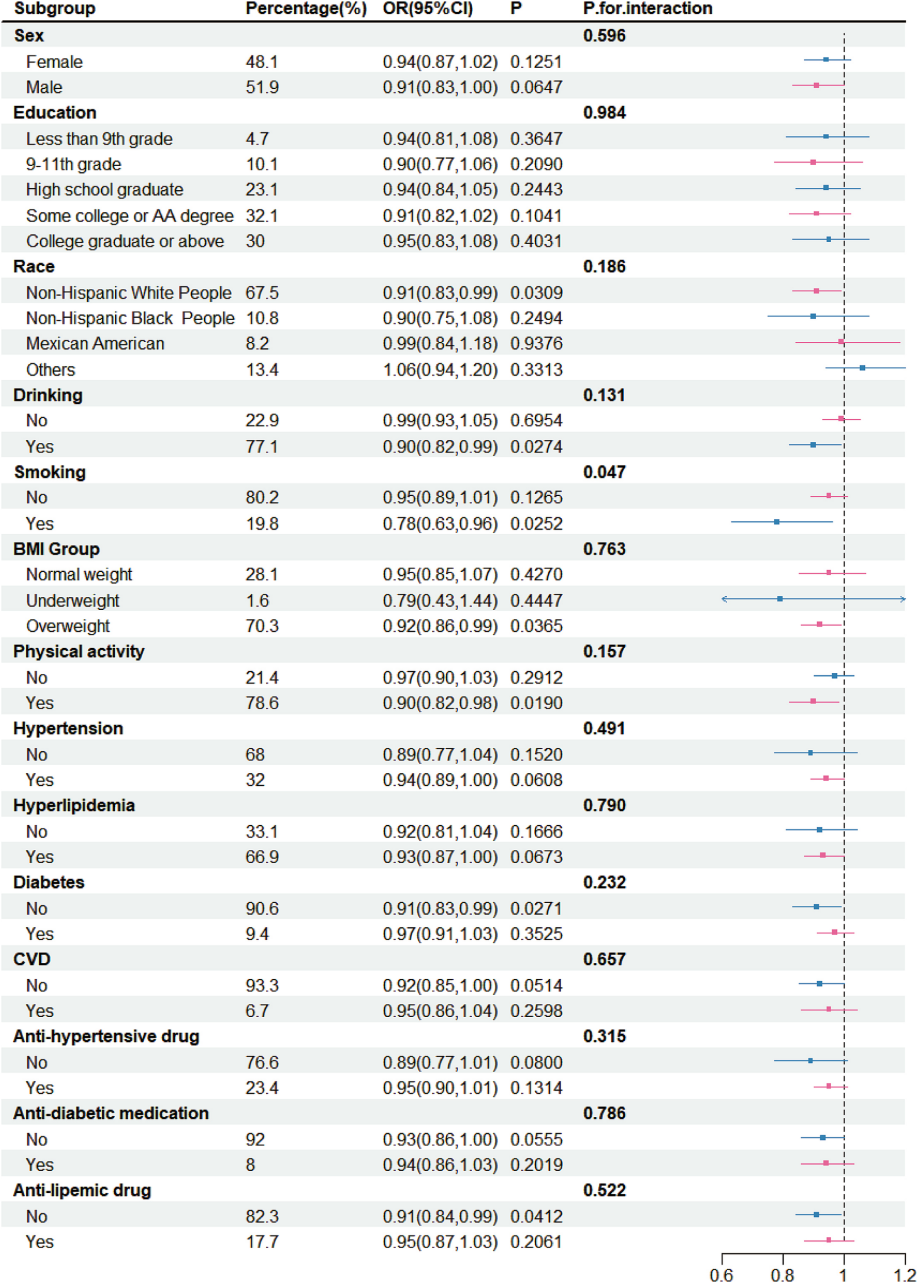
Subgroup analysis for the association between intake of fermented dairy products and stroke, weighted.

The above results showed that both total intake and yogurt intake were negatively correlated with stroke risk. Therefore, detailed subgroup analyses conducted. In these analyses, both total fermented dairy intake (50 g/day) and yogurt intake (50 g/day) were treated as continuous variables. For the relationship between total intake and stroke risk (Figure 3), no significant differences were observed across different sex groups, education groups, race groups, BMI groups, drinker groups, CVD groups, hypertension groups, diabetes groups, and hyperlipidemia groups. However, in certain specific populations, the ingestion of fermented dairy products was related to a lowered risk of stroke, and this relationship was statistically significant. These populations included White people (OR = 0.91, 95% CI: 0.83–0.99), drinkers (OR = 0.90, 95% CI: 0.82–0.99), smokers (OR = 0.78, 95% CI: 0.63–0.96), overweight individuals (OR = 0.92, 95% CI: 0.86–0.99), those with physical activity (OR = 0.90, 95% CI: 0.82–0.98), those without CVD (OR = 0.92, 95% CI: 0.85–1.00), those without diabetes (OR = 0.90, 95% CI: 0.83–0.98), those without anti-diabetic medication (OR = 0.93, 95% CI: 0.86–1.00) and those without anti-lipemic drug (OR = 0.91, 95% CI: 0.84–0.99). However, we found ferment milk may benefit the smoker more (*p* for interaction = 0.047).

**Figure 4:**
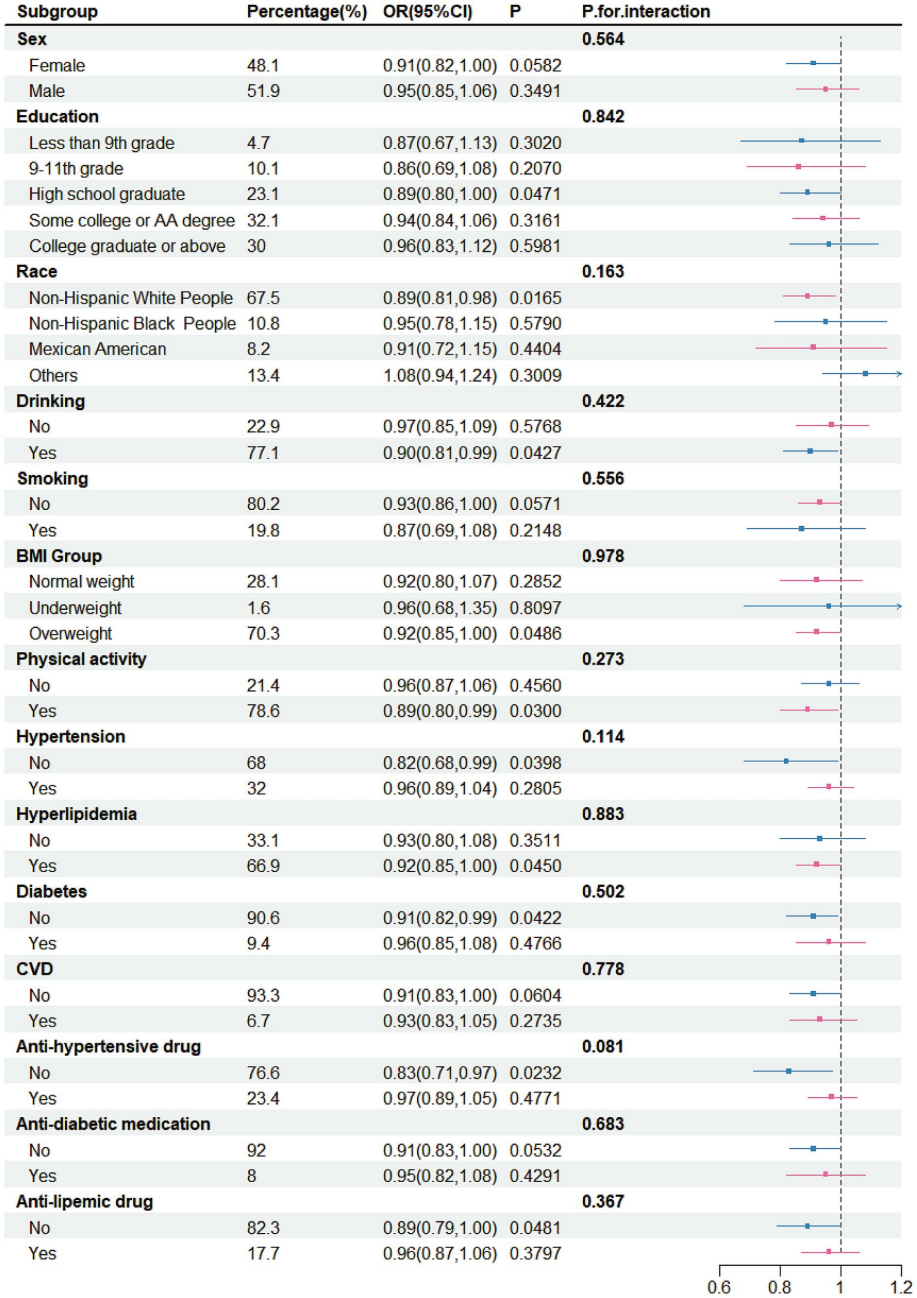
Subgroup analysis for the association between yogurt intake and stroke, weighted.

There was no significant correlation between yogurt intake and stroke risk across different subgroups (Figure 4). However, a negative correlation was observed in certain specific groups, including female (OR = 0.91, 95% CI 0.82–1.00), participants with high school education (OR = 0.89, 95% CI 0.80–1.00), White people (OR = 0.89, 95% CI: 0.81–0.98), drinkers (OR = 0.90, 95% CI: 0.81–0.99), non-smokers (OR = 0.93, 95% CI: 0.86–1.00), individuals who were overweight (OR = 0.92, 95% CI: 0.85–1.00), those with physical activity (OR = 0.89, 95% CI: 0.80–0.99), those without hypertension (OR = 0.82, 95% CI: 0.68–0.99), individuals with hyperlipidemia (OR = 0.92, 95% CI: 0.85–1.00), those without diabetes (OR = 0.91, 95% CI: 0.82–0.99), those without anti-hypertensive drug (OR = 0.83, 95% CI: 0.71–0.97), those without anti-diabetic medication (OR = 0.91, 95% CI: 0.83–1.00) and those without anti-lipemic drug (OR = 0.89, 95% CI: 0.79–1.00).

### 3.5 The association between fat content in fermented dairy products and stroke

This analysis was performed separately in three consumption subgroups across three distinct models: yogurt consumers, buttermilk consumers, and cheese consumers (Table 6). Due to the insufficient number of individuals consuming buttermilk, no statistical results were obtained, thus the data were not presented in the table. Results showed that the fat content in both yogurt and cheese was not associated with stroke risk (*p* > 0.05).

**Table 6:** Association between fat content in fermented dairy products and stroke, weighted.

Exposure	Model I OR (95% CI)	*p*	Model II OR (95% CI)	*p*	Model III OR (95% CI)	*p*
Fat content in yogurt	0.93 (0.83, 1.03)	0.155	0.94 (0.83, 1.06)	0.279	0.91 (0.81, 1.03)	0.127
Fat content in cheese	0.99 (0.97, 1.01)	0.496	1.01 (0.99, 1.02)	0.436	1.01 (0.99, 1.02)	0.464

Model I: no adjustment. Model II: adjust for age, sex, race. Model III: adjust for age, sex, race, PIR, education, smoking, drinking, BMI, physical activity, diabetes, hypertension, hyperlipidemia, CVD, anti-hypertensive drug, anti-lipemic drug, and anti-diabetic medication and cow milk intake. PIR, poverty income ratio; BMI, body mass index; CVD, cardiovascular disease; OR, odds ratio; CI, confidence interval.

## 4 Discussion

This research, based on an in-depth analysis of data from the NHANES database (2007–2018), revealed that fermented dairy products may potentially mitigate stroke risk. Notably, it was yogurt, as opposed to cheese and buttermilk, that exhibited a remarkably negative correlation with stroke incidence. Specifically, among the people who consumed yogurt, the incidence of stroke was the lowest. The findings demonstrated that for each incremental 50-gram increase in the daily intake of fermented dairy products, the risk of stroke was diminished by 7%. Moreover, the optimal range for minimizing the stroke risk lay within the daily consumption of 0–38.3 grams of fermented dairy products. Similarly, in the case of yogurt consumption, a 50-gram increment in daily intake corresponded to a 7% reduction in stroke risk. However, consuming more than 170 g of yogurt daily could reduce the risk of stroke. Conversely, the results of this study did not indicate any significant correlation between cheese, buttermilk, and the risk of stroke. In conclusion, having a sufficient quantity of fermented dairy products, particularly yogurt, every day can help American people avoid stroke by promoting a balanced diet.

Diet is a modifiable factor in stroke prevention, and dairy products are an essential component of a wholesome diet. Previous studies have yielded results similar to ours. A prospective cohort study (29) reported a trend toward an inverse association between fermented dairy consumption and the risk of stroke (*p* = 0.07). An investigation of a cohort in the Netherlands showed that the ingestion of yogurt was notably linked to a lower risk of stroke in both genders (30). Another Dutch study (31) involving 36,886 participants showed that full-fat yogurt, compared with other dairy products, was related to a decreased likelihood of stroke (HR = 0.34–0.37). Similarly, observations from nine European countries indicated that a daily ingestion of fermented dairy products showed an inverse relationship to the risk of ischemic stroke (yogurt: 100 g/day, HR = 0.91, 95% CI: 0.85–0.97; cheese: 30 g/day, HR = 0.88, 95% CI: 0.81–0.97) (32). Additionally, a Danish cohort study including 55,211 participants found that replacing other dairy products with full-fat fermented dairy was associated with a lower incidence of ischemic stroke (33). However, these studies do not appear to elucidate the causal relationship between fermented dairy products and stroke risk. In summary, both previous studies and this research provide a common suggestion that the intake of fermented dairy products, particularly yogurt, may reduce the risk of stroke. Moreover, while moderate consumption is generally appropriate for total fermented dairy intake, a daily yogurt intake of more than 170 g is required to achieve a protective effect.

However, in these results, the relationship between cheese and stroke was not statistically significant. Moreover, when compared with those who did not consume cheese, the negative correlation disappeared after adjusting for covariates. This result may be related to the insufficiently detailed classification of cheese in the database. Due to the diversity of food types nowadays, perhaps not all the consumed cheese was included in the statistics. Meanwhile, cheese, with its characteristics of high fat and high sodium, is considered a food that is not conducive to cardiovascular and cerebrovascular health (34, 35). The results of some studies also show that the correlation between cheese and stroke is not statistically significant (RR = 0.97, 95% CI: 0.94–1.01) (36). Regarding buttermilk, only 74 people (0.27%) in the population covered by this study consumed it. The extremely small sample size makes the relationship between buttermilk and stroke not particularly clear. However, as a by-product of the butter manufacturing process, buttermilk is characterized by its low fat and low salt content. The milk fat globule membrane (MFGM), which is abundant in buttermilk, has been verified to have anti-inflammatory properties and the potential to reduce cholesterol levels (37), and can restore the normal flora structure of mice with dysbacteriosis (38). MFGM, a trilayer structure composed of proteins and polar lipids (phospholipids and sphingolipids), tightly encapsulates the triglyceride core (fat globule) as a natural protective layer. Sphingolipids and bovine phospholipids within MFGM can reduce serum cholesterol levels and alleviate hepatic lipid accumulation by decreasing intestinal cholesterol absorption and regulating hepatic gene expression related to lipid metabolism (39, 40). Moreover, buttermilk processed by certain special techniques may even substitute for milk (41). Evidently, it is also a potentially healthy beverage.

In the analysis of baseline data, there was no difference in the gender distribution between stroke and non-stroke participants. This may be related to the relatively small sample size. However, some studies have also shown that the gender-specific differences in the prevalence of stroke have been decreasing year by year (42). It is widely recognized that alcohol consumption is a factor in stroke risk. However, the results of this study show that a significant number of participants who did not suffer from stroke had a habit of drinking alcohol. Similarly, some studies have shown that moderate alcohol consumption and the intake of certain types of alcoholic beverages could be related to a reduced risk of stroke (43, 44). Therefore, the relationship between alcohol consumption and stroke risk remains a topic of debate.

In the subgroup analysis, correlations are not universal because the sample sizes of each subgroup are relatively small. When the total intake was selected for analysis, a negative correlation between stroke and it was observed among White people, drinkers, smoker, overweight individuals, those with physical activity, those without CVD, those without diabetes, those without anti-diabetic medication and those without anti-lipemic drug. After that, subgroup analyses were also conducted on the relationship between yogurt and stroke. The negative correlation was not present in any of the subgroups. However, there were some populations that might benefit, such as participants with a high—school education, White people, drinkers, non-smokers, overweight individuals, those with physical activity, those without hypertension, individuals with hyperlipidemia, those without diabetes, those without anti-hypertensive drug, those without anti-diabetic medication and those without anti-lipemic drug. This suggests that healthcare workers should provide targeted prevention strategies for specific populations when formulating preventive policies. The above-mentioned population should pay more attention to the intake of fermented dairy products.

Fermented dairy product, as a traditional food, has its health benefits well-documented by extensive research, providing an important research entry point for exploring the potential mechanisms underlying the association between fermented milk and stroke risk. The gut-brain axis is a complex bidirectional signaling system connecting the gastrointestinal tract and the brain (45). This connection is mediated through the synergistic actions of the Central Nervous System (CNS), the Enteric Nervous System (ENS), and the gut microbiota. The ENS is an intricate system composed of neurons and glial cells embedded within the intestinal wall, regulating most gastrointestinal functions. Furthermore, the ENS establishes bidirectional communication with the CNS via autonomic nerves and primary afferent nerves originating from the gastrointestinal tract (46). In this process, it is the gut microbiota that consistently plays a crucial role. Probiotics are collectively defined as live microorganisms that, when ingested in appropriate amounts, exhibit biological activity, promote ecological balance of the intestinal microbiota, and confer health benefits to the host (47). Fermented dairy products are primary food carriers for probiotics (48), and their stroke-preventive function is inseparable from the role of these beneficial microorganisms. Under normal physiological conditions, the gut microbiota sustains a dynamic equilibrium, forming a gut barrier that supports host health (49). When the abundance and diversity of gut microbiota are reduced, it may directly trigger a stroke. A review of 14 clinical studies found that stroke patients had lower gut microbiota diversity compared to healthy individuals (50). However, this effect is relatively weak, and the impact of gut microbiota dysbiosis on stroke occurrence is more likely mediated through influencing risk factors (7, 51). Current research indicates that obesity (52), diabetes (53), and hypertension (54) are risk factors directly associated with gut microbiota imbalance. Studies have shown that one serving of yogurt contains approximately 10 million CFUs (colony-forming units) of bacteria (11),with high diversity (55), including *Bifidobacterium lactis*, *Lactobacillus rhamnosus*, and *Streptococcus thermophilus*. And most of these bacteria are beneficial to human health. For instance, *Lactobacillus rhamnosus* can inhibit inflammation (56), and *Bifidobacterium lactis* reduces BMI (57). The intake of fermented dairy products can increase the number of probiotics (58), improve the gut microenvironment, and produce a series of beneficial effects on the human body, thereby influencing risk factors (59) and ultimately reducing the incidence of stroke.

In addition to affecting the homeostasis of the gut microbiota, probiotics can also influence the metabolites or intermediate products of the gut microbiota, which may be another mechanism by which fermented dairy products prevent stroke. Certainly, this preventive effect is also achieved by influencing risk factors. These substances include short-chain fatty acids (SCFAs) and conjugated linoleic acid. Conjugated linoleic acid is widely present in dairy products, but the lactic acid bacteria and bifidobacterium in yogurt can increase its content (60), thereby further enhancing its effects of reducing blood pressure, blood lipids, and body weight (61). Similarly, these bacteria can ferment some dietary fibers, thus increasing the content of metabolites, and this is how SCFAs are increased (62). A literature review found that fermented milk can significantly reduce cholesterol compared with non-fermented milk, and this has been verified both in humans and animals (63). This may be related to the role of short-chain fatty acids in inhibiting cholesterol synthesis in the liver. In addition, experiments have found that SCFAs can promote the secretion of peptide YY by intestinal endothelial cells, thereby increasing insulin secretion and achieving the effect of lowering blood glucose (64). Moreover, an animal experiment has found that SCFAs can directly act on the nerve endings in the lamina propria of intestinal mucosal epithelial cells, thereby activating the afferent nerve fibers of the vagus nerve and affecting blood pressure regulation (65). Another factor related to stroke is the inhibitory neurotransmitter—GABA. Lactobacillus rhamnosus and Bifidobacterium have been proven to produce this neurotransmitter in the intestine (66), and yogurt and cheese themselves are foods rich in GABA (67, 68). Different from other factors, GABA can also regulate emotions, and the influence of emotional issues on stroke occurrence has garnered significant attention nowadays. Multiple meta-analyses and systematic reviews (69, 70) have shown that negative emotions (depression) are positively correlated with stroke. GABA can relieve depression (71), thereby reducing the impact of emotional problems on stroke risk. At the same time, GABA has also been reported to have the effect of lowering blood pressure (72). Certainly, existing studies have indeed confirmed that fermented dairy products can prevent or improve these risk factors (73–77). In conclusion, probiotics and their derivatives significantly contribute to stroke prevention through fermented dairy products.

In addition, we hypothesize that the high calcium content in fermented dairy products is another important factor in stroke prevention. As early as 1931, it was discovered that yogurt contains higher levels of calcium than milk, and the presence of probiotics facilitates calcium absorption. Studies have shown that calcium intake is associated with a lower prevalence of stroke. Zhu et al. analyzed the dietary intake of 6,411 Chinese individuals and found that calcium intake appeared to correlate with a reduced likelihood of stroke (HR = 0.53, 95% CI: 0.29–0.96) (78). A 14-year follow-up study (14) revealed that low calcium intake increased the stroke risk in adult women, while those with high calcium intake had a relative stroke risk of 0.69 (95% CI: 0.50–0.95). This inverse correlation was more pronounced for calcium obtained from dairy sources (dairy: RR = 0.68, 95% CI: 0.50–0.94; non-dairy: RR = 0.82, 95% CI: 0.58–1.16). Tian et al. also obtained similar results through a meta-analysis of several prospective cohort studies (79). Similarly, a study focused on men also found that dietary calcium intake can lower stroke incidence (80). In summary, dietary calcium can reduce stroke risk to some extent, which may be related to its ability to influence stroke risk factors. For example, calcium can directly affect the contraction and relaxation of vascular smooth muscle, thereby regulating blood pressure (81). Additionally, calcium can lower cholesterol levels in the blood (82). This explains why calcium-rich fermented dairy products can help prevent stroke.

Finally, the preventive effect of fermented milk against stroke may be related to its anti-inflammatory properties. Chronic inflammation is recognized as a key factor in stroke occurrence (83). Inflammatory factors (such as interleukins and tumor necrosis factor) produced by chronic inflammation can alter the state of vascular endothelium, activate platelets, inhibit the decomposition of fibrinogen, promote a hypercoagulable state, form blood clots, and increase the risk of stroke (84). Fortunately, diet can modulate the systemic inflammatory response. Moreover, an analysis of the NHANES database reveals a positive correlation between the dietary inflammatory index and stroke risk, indicating that an anti-inflammatory diet can reduce the risk of stroke (25). Notably, fermented dairy products have demonstrated anti-inflammatory effects (10). For instance, a 9-week randomized controlled trial on healthy premenopausal women showed that compared with women who consumed soy pudding daily, women who consumed 226 grams of low-fat yogurt daily for 9 weeks had a decrease in the marker of chronic inflammation, IL-6, in their bodies (85). Additionally, some studies have also shown that Bifidobacterium can significantly reduce the levels of tumor necrosis factor-*α* and IL-6 in patients (57). Collectively, these findings suggest that the preventive effect of fermented dairy products against stroke is likely mediated by their ability to alleviate the pro-inflammatory response.

This research elucidated the connection between overall consumption of fermented dairy products and stroke risk, alongside the links between specific fermented dairy items (yogurt, cheese, and buttermilk) and stroke incidence. Additionally, we established the dose–response relationships for these associations: stroke risk decreased by 7% for every 50 g increase in total fermented dairy intake. Notably, yogurt intake was associated with an even greater reduction in stroke risk, with each 50 g increase lowering it by 7%. No such associations were observed for cheese or buttermilk. However, our study has certain limitations. First, stroke and confounding variables were determined based on participants recall, which may introduce recall bias. The survey questionnaire also did not specify the type of stroke. Second, as this was a cross-sectional study, we could not establish causality between fermented dairy intake and stroke. Third, the intake of dairy products was based on participants’ recall, which may be inaccurate. Furthermore, due to the diversification of modern food products, some emerging fermented dairy products may not have been included in the database. Participants may have consumed fermented dairy products in other foods that were not accounted for, potentially affecting the results. Additionally, the dietary intake data represented only a two-day average for participants, which might not accurately represent their long-term dietary patterns. Nevertheless, studies have shown that this method of data collection is representative (86). Furthermore, the NHANES database did not analyze statistically the participants’ background of daily dairy consumption or their dietary patterns, which may have influenced the results. Fourth, non-dairy fermented foods might affect stroke occurrence through similar pathways. However, due to challenges in accurately quantifying their intake, they were not included as covariates, potentially introducing errors. Finally, given the complexity of stroke occurrence, there may still be unconsidered confounding factors in the NHANES database, such as family history of stroke.

## 5 Conclusion

This research revealed a negative correlation between fermented dairy products and stroke among adult Americans, particularly with yogurt. Consuming fermented dairy products in appropriate amounts daily may lower stroke risk. However, no significant correlation was observed between cheese, buttermilk, and stroke. These findings can provide recommendations for adult Americans to prevent stroke. Nonetheless, numerous prospective cohort studies remain necessary to confirm these findings and elucidate the causal relationship.

## Data Availability

Publicly available datasets were analyzed in this study. This data can be found at: https://www.cdc.gov/nchs/nhanes/.

## Footnotes

^1^ https://www.r-project.org
